# Supplementary figures and images for: Genome-wide SNPs reveal the drivers of gene flow in an urban population of the Asian Tiger Mosquito, Aedes albopictus
Source: PLoS Negl Trop Dis. 2017 Oct 18;11(10):e0006009. doi: 10.1371/journal.pntd.0006009 (PMC5662242; doi:10.1371/journal.pntd.0006009)

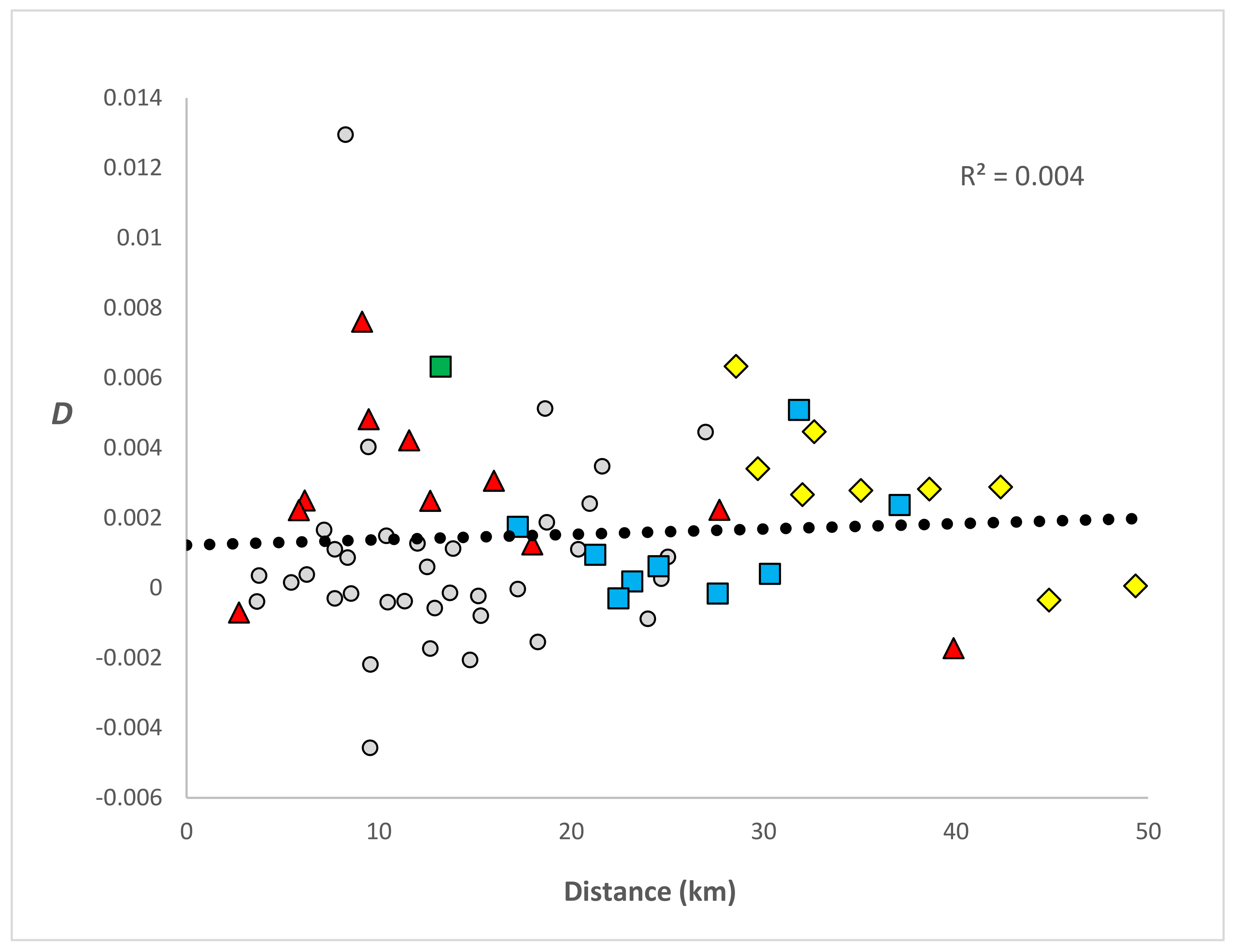

Supplement: S1 Fig — Colour codes are the same as in Fig 3. No overall trend of IBD was observed. (TIF) [file pntd.0006009.s003.tif]
